# Substantial kelp detritus exported beyond the continental shelf by dense shelf water transport

**DOI:** 10.1038/s41598-023-51003-5

**Published:** 2024-01-08

**Authors:** Mirjam van der Mheen, Thomas Wernberg, Charitha Pattiaratchi, Albert Pessarrodona, Ivica Janekovic, Taylor Simpkins, Renae Hovey, Karen Filbee-Dexter

**Affiliations:** 1 School of Biological Sciences and UWA Oceans Institute, University of Western Australia, Perth, WA Australia; 2 Institute of Marine Research, Nye Flødevigveien 20, His, 4817 Norway; 3 Oceans Graduate School and UWA Oceans Institute, University of Western Australia, Perth, WA Australia

**Keywords:** Ecosystem services, Marine biology, Physical oceanography, Climate-change mitigation

## Abstract

Kelp forests may contribute substantially to ocean carbon sequestration, mainly through transporting kelp carbon away from the coast and into the deep sea. However, it is not clear if and how kelp detritus is transported across the continental shelf. Dense shelf water transport (DSWT) is associated with offshore flows along the seabed and provides an effective mechanism for cross-shelf transport. In this study, we determine how effective DSWT is in exporting kelp detritus beyond the continental shelf edge, by considering the transport of simulated sinking kelp detritus from a region of Australia’s Great Southern Reef. We show that DSWT is the main mechanism that transports simulated kelp detritus past the continental shelf edge, and that export is negligible when DSWT does not occur. We find that 51% per year of simulated kelp detritus is transported past the continental shelf edge, or 17–29% when accounting for decomposition while in transit across the shelf. This is substantially more than initial global estimates. Because DSWT occurs in many mid-latitude locations around the world, where kelp forests are also most productive, export of kelp carbon from the coast could be considerably larger than initially expected.

## Introduction

Global anthropogenic greenhouse gas emissions continue to increase and have already caused widespread damage to nature and humans^1^. Current global mitigation policies are insufficient to limit warming below 2 °C, and there is a need to find new ways to mitigate emissions. The ocean plays a key role in the global carbon cycle^2^, and so-called “blue carbon” coastal ecosystems are responsible for sequestering large amounts of organic carbon^3^. Protecting and restoring these ecosystems is one of the most cost-effective strategies to increase sequestration in the ocean and avoid more carbon emissions^4^ and can mitigate up to 3% of global emissions^5^.

Vegetated coastal ecosystems such as mangroves, saltmarshes, and seagrasses sequester globally significant amounts of blue carbon via burial in sediments^3,6^. Seaweeds were initially not considered to contribute to blue carbon storage, because most seaweeds grow in rocky habitats where in situ carbon burial is precluded. However, seaweed ecosystems are by far the most globally widespread and productive vegetated coastal habitat, contributing around 20% of the carbon fixed in the coastal ocean^7,8^. Seaweed carbon fluxes are overwhelmingly dominated by seaweed forests such as those formed by kelp (Order Laminariales), which ultimately release most carbon that they fix either in dissolved form or as particulate detritus^9^. Some of this carbon can then be transported from the coast into depths beyond the continental shelf where there is no exchange with the atmosphere for significant periods of time (defined as “deep sea”)^10^.

**Figure 1:**
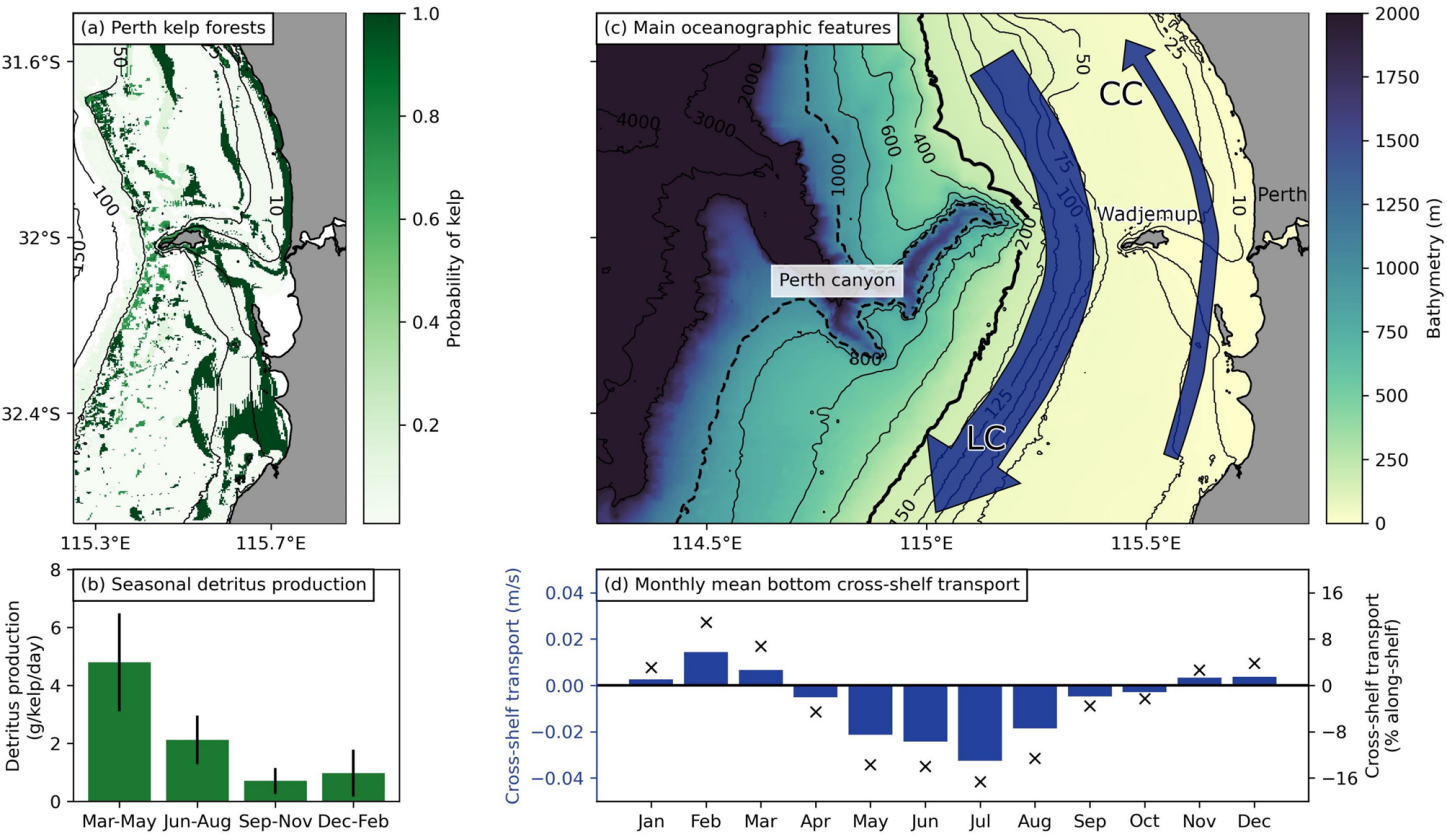
Overview of the Wadjemup continental shelf (WCS) region. **(a)** Map showing the probability of kelp in the Perth region (see “Methods” section). **(b)** Seasonal variation of kelp detritus production, based on data from de Bettignies et al.^13^. **(c)** Map showing the main topological and oceanographic features of the WCS region. Background colors show the bathymetry based on data from Geoscience Australia^14^, with the 200 m depth contour highlighted as the continental shelf edge. The Perth Canyon is located west of Wadjemup. A schematic representation of the southwards flowing Leeuwin Current (LC) and the northwards flowing Capes Current (CC) are shown in blue arrows. The LC is the predominant ocean surface current and extends to approximately 200 m depth^15^. The CC is seasonal and driven by strong southerly seabreezes in the warmer months^16^. **(d)** Monthly mean bottom cross-shelf transport along the 100 m depth contour (defined as being directed perpendicular to the depth contour), based on CWA-ROMS data for 2017 and averaged over the bottom three model layers. Negative values indicate transport away from the coast and towards the open ocean, and positive values indicate transport towards the coast. Blue bars show the monthly mean bottom cross-shelf velocities in m/s (between 0.002 and 0.03 m/s), black crosses indicate the cross-shelf velocity as a percentage of the monthly mean bottom along-shelf velocity (between 2 and 17% of the mean along-shelf velocity, which itself is between 0.08 and 0.2 m/s). Note that the velocities shown here are used to illustrate the seasonal mean cross-shelf bottom dynamics but are not used to derive any results.

Although this is one of the main proposed pathways for kelp forests to contribute to carbon sequestration^10^, it is still unknown how much kelp detritus is successfully exported from the coast into the deep sea. In fact, alongshore ocean currents dominate the flow field in the coastal ocean, but cross-shelf currents are needed to transport kelp detritus beyond the continental shelf and into the deep sea. These cross-shelf currents are typically much weaker than alongshore currents^11^ (Fig. 1d) and are caused by a combination of different processes that vary at each time and location, making them notoriously difficult to study^12^. As a result, understanding cross-shelf exchange is still one of the central knowledge gaps in coastal physical oceanography. Consequently, key remaining questions center on if, how, and how much kelp detritus may be exported from the coast and into the deep sea.

A mechanism that clearly leads to cross-shelf transport is dense shelf water transport (DSWT). DSWT is a density-driven offshore flow along the seafloor that arises from increased water density along the coast as a result of cooling and evaporation of surface water^17^. DSWT occurs in multiple mid-latitude locations (approximately between 30° and 60° latitude) around the world^18,19^, which is also where kelp forests are most productive^8^. DSWT is associated with relatively rapid cross-shelf transport along the seafloor, and so could be a strong mechanism to transport negatively buoyant (sinking) kelp detritus across the continental shelf and into the deep sea.

DSWT could be a particularly relevant transport mechanism for kelp detritus along Australia, where it occurs along all of the continental shelves during the colder months^20^. Australia is also home to the Great Southern Reef: an extensive system of interconnected kelp forests spanning over 8000 km^21^ along the southern margin of the continent. The dominant kelp species (golden kelp—*Ecklonia radiata*) is negatively buoyant, which means that its detritus would be transported along the seafloor. To test how kelp detritus may be transported by DSWT, we use particle tracking simulations to determine the transport of *Ecklonia radiata* detritus across the Wadjemup (Rottnest) continental shelf (WCS), a sub-region of the Great Southern Reef.

### Wadjemup continental shelf: kelp forests and oceanographic setting

Kelp forests on the WCS extend from shallow reefs ($<10$ m) along the coast to deeper reefs ($\sim$ 50 m) further out on the continental shelf (Fig. 1a). Up to 50 m, the continental shelf slopes gently downwards, but rapidly increases in depth from 50 m until the continental shelf edge at 200 m. A shallower region extends between the Perth coastline and Wadjemup (Rottnest Island), approximately 20 km west of Perth. The Perth canyon is located roughly 20 km west of Wadjemup, and drops to depths of $>6000$ m (Fig. 1c).

Kelp in this region produces detritus year-round, but detrital production increases from March until August, peaking from March to May^13^ (Fig. 1b). Once released into the coastal ocean, detritus is transported by ocean currents and remineralized by bacteria and detritivores. Because the dominant species in this region (golden kelp—*Ecklonia radiata*) is negatively buoyant, kelp detritus is transported by bottom ocean currents. The strength and direction of ocean currents depends on the season. In the following paragraphs, we describe the ocean dynamics and how they are likely to affect the transport of detritus from March to August.

Peak production of kelp detritus starts in March, which is one of the hottest months of the year. During this time, the local dynamics are dominated by seabreezes, which are among the strongest in the world, and frequently result in southerly winds $>15$ m/s during the afternoon^22,23^. As a result of these strong southerly winds, the southward Leeuwin Current (LC), the main boundary current along Western Australia^15^ (Fig. 1c), is relatively weak and located further offshore. These winds also drive the seasonal Capes Current (CC), which flows northward inshore of the LC^16^ (Fig. 1c). The mean cross-shelf transport along the seafloor is directed towards the coast (Fig. 1d) in March. Because of this, no significant export of kelp detritus beyond the continental shelf is expected. Instead, kelp detritus will predominantly be transported northwards by the CC while remaining on the continental shelf.

**Figure 2:**
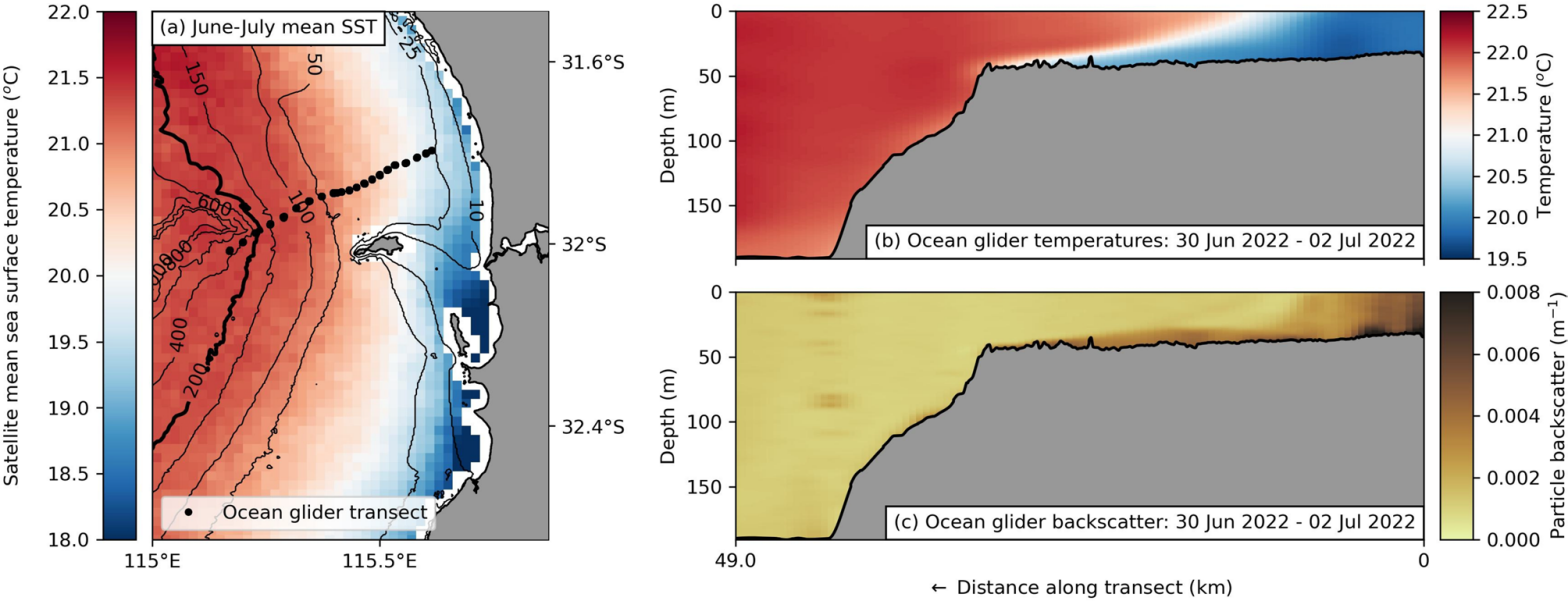
Dense shelf water transport (DSWT) suitable conditions during winter cooling and transects of a DSWT event. **(a)** Mean sea surface temperature during June and July from 2012–2022 using the gridded, multi-sensor, multi-swath day and night monthly averaged AVHRR IMOS-GHRSST L3S dataset. A band of cooler water along the coast during these months enables the formation of DSWT. Ocean glider measurements from IMOS along a transect (shown with black dots in (**a**)) during a mission from the 30th of June until the 2nd of July 2022, showing: **(b)** ocean temperatures; and **(c)** backscatter (a proxy for suspended matter) during a dense shelf water transport event. Note that the backscatter measures all suspended matter and is likely a mixture between sediment and organic material (including kelp detritus). It is shown here to illustrate the potential for DSWT to transport suspended material across the continental shelf, but is not used to measure the transport of kelp detritus.

During April and May there is a transition into cooler weather and low winds. June and July are the coldest months of the year, with many winter storms. Although June and July are also the wettest months, the annual mean evaporation rate ($\sim$ 2.5 m) in this region is far greater than the rainfall ($\sim$ 730 mm)^24^. As a result, differential heat loss during these cold months creates a cross-shelf density gradient with a band of colder, denser water forming along the coast (Fig. 2a). This horizontal density gradient creates DSWT (previously defined as “dense shelf water cascades”^20,24^). DSWT can be easily recognized in cross-shelf transects as wedges of denser water (in this region: colder water) protruding along the shelf below the lower density (warmer) water. These events have frequently been observed on the WCS by ocean gliders^20,24^ (Fig. 2b for a recent example) and can be simulated using regional ocean models^24^ (Fig. S1). DSWT provides an effective mechanism to transport suspended material past the continental shelf edge (Fig. 2c as an example). As the occurrence of DSWT increases during the colder months^20^, the mean cross-shelf bottom transport is directed offshore starting from May and peaking in July (Fig. 1d). In June and July, the weather conditions are such that storms can re-suspend detritus and the calmer periods between storms allow for DSWT. Although mixing due to strong winds can disrupt the formation of DSWT, onshore winds, frequent during winter storms in June and July, enhance DSWT and associated offshore transport^20^. We therefore expect kelp detritus to be exported past the continental shelf edge during these colder seasons.

Final substantial detritus production occurs in August. During this time, DSWT still occurs, and the mean transport along the seafloor is still directed offshore (Fig. 1d), but this decreases as the warmer months start to arrive again. During the remaining months (September until February) detrital production is much lower. During September and October the mean transport is still (weakly) offshore. From November to February the mean transport along the seafloor is directed onshore. We do not expect these months to contribute much to the export of kelp detritus, both because of limited detrital production and limited offshore transport.

## Results

**Figure 3:**
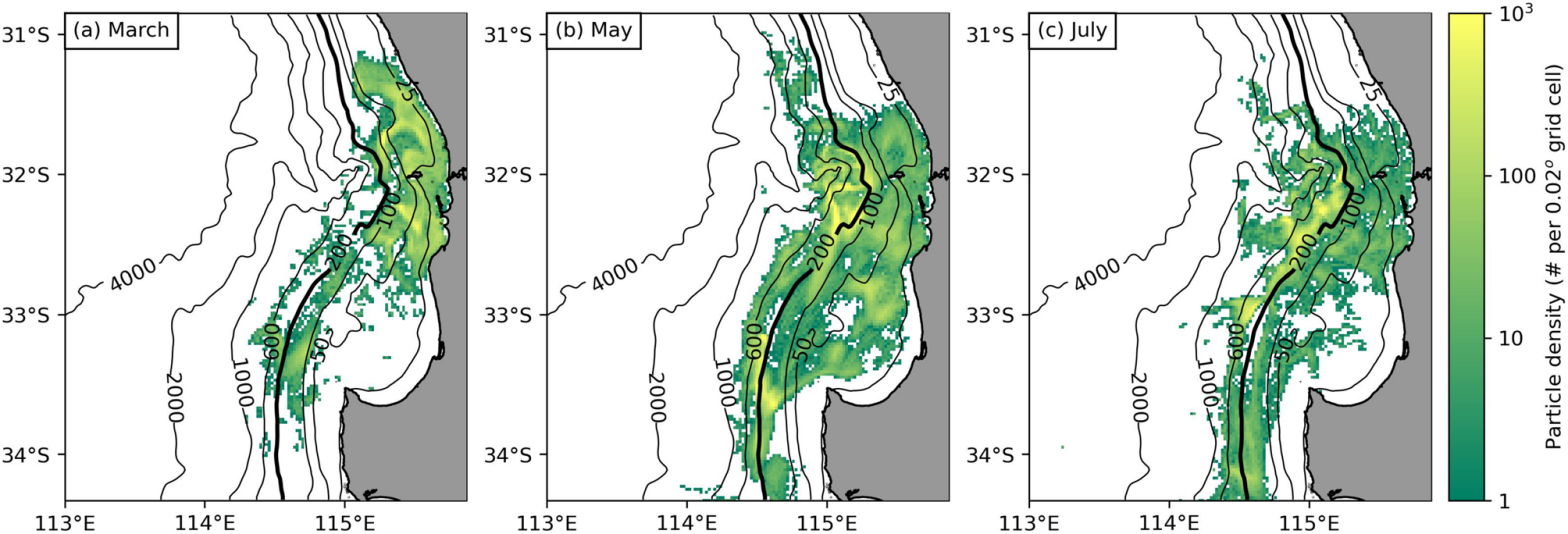
Near-bed density maps of simulated (non-decomposing) kelp detritus during peak detrital production months released from the Perth region (Fig. 1a) at the end of: **(a)** March, one of the hottest months of the year; **(b)** May, when cooler weather begins; and **(c)** July, one of the coldest and wettest months of the year. The thick black 200 m contour line highlights the continental shelf edge. During March hardly any simulated detritus was transported past the continental shelf edge. In contrast, during the cooler months of May and July, when dense shelf water transport occurred, simulated detritus was consistently transported past the shelf edge.

During the peak detrital production period from March until August, our particle tracking simulation results showed that hardly any simulated kelp detritus was transported past the continental shelf during March (Fig. 3a). This was expected because the dynamics are dominated by (upwelling-favorable) seabreezes resulting in bottom cross-shelf transport towards the coast (which is the flow needed to balance the net offshore transport in the wind-driven surface Ekman layer) during this time. In contrast, during the colder months of April and May (Fig. 3b), as well as June and July (Fig. 3c) substantial amounts of simulated detritus were exported across the continental shelf, which was expected because of DSWT during these months.

**Figure 4:**
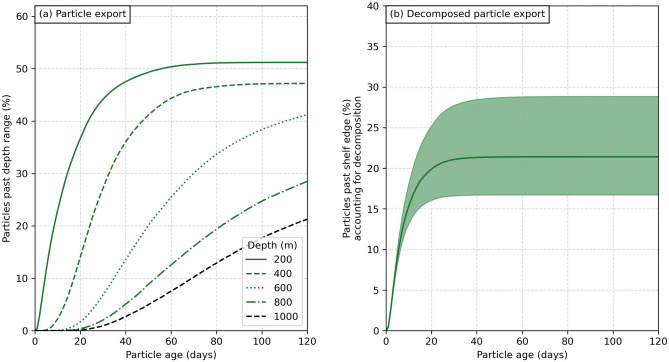
Export of simulated kelp detritus for a full year. **(a)** Percentage of (non-decomposing) kelp detritus exported past different depth ranges from 200 m (continental shelf edge) up to 1000 m as a function of the detritus’ age after being released into the coastal ocean environment. **(b)** Percentage of detritus exported past the continental shelf edge at 200 m, when accounting for the decomposition of kelp detritus while in transit along the shelf. The dark green line shows the export percentage based on the mean decomposition rate determined by Simpkins et al.^25^. The lighter green band around this line shows the result for the mean ± one standard deviation of the decomposition rate.

Overall for an entire year, we found that 51% of simulated kelp detritus was transported past the continental shelf edge at 200 m depth, with decreasing percentages exported past deeper ranges (Fig. 4a). However, as kelp detritus drifts, it decomposes. Accounting for this using locally-derived decay rates of the dominant kelp species (golden kelp—*Ecklonia radiata*; $k = 0.075 \pm 0.031$ per day, equivalent to a half-life of $t_{1/2} = 9.2 \pm 2.7$ days^25^), we found that 21% (range 17–29%) of simulated decomposing kelp detritus is exported past the continental shelf edge (Fig. 4b). Here, we assume that the decay rate measured by Simpkins et al.^25^ at 10, 20, and 50 m depth is valid on the continental shelf. We do not estimate the export of decomposing kelp detritus past deeper ranges below 200 m, because the decomposition rate may be substantially different^26^.

### Export mechanism

**Figure 5:**
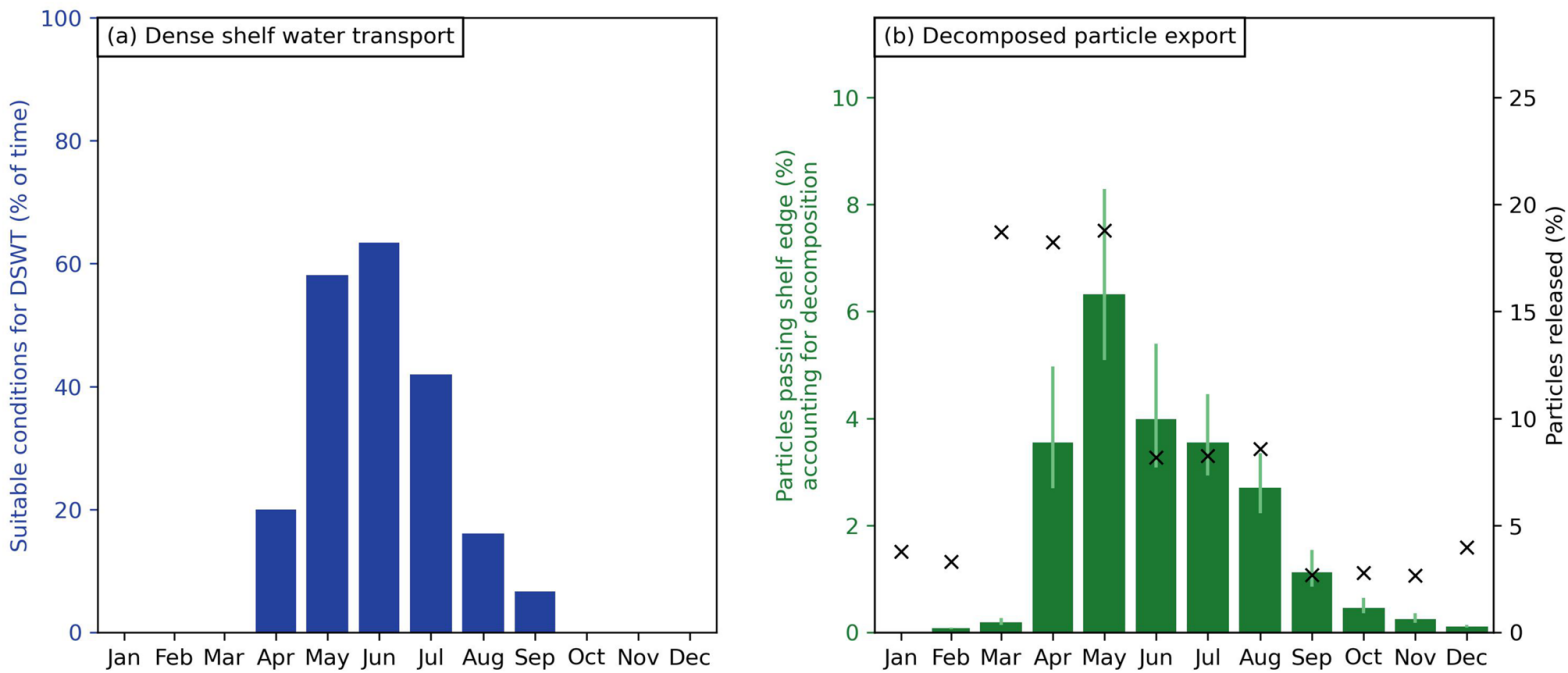
Monthly variation in environmental conditions suitable for dense shelf water transport (DSWT) and exported simulated kelp detritus. **(a)** Suitable environmental conditions for the formation of DSWT, as a percentage of time for each month (see Fig. S8 for a breakdown of the different conditions required for DSWT to form). **(b)** Percentage of total decomposing kelp detritus exported past the continental shelf edge per month (green bars), compared to the percentage of detritus released each month in our simulation (black crosses). Note that the sum of the monthly percentages adds up to the export percentage for a full year of 21%.

We expected that the majority of kelp detritus exported beyond the continental shelf was due to DSWT during the colder months. There is indeed a clear seasonal difference in the amount of simulated decomposing kelp detritus that was exported beyond the continental shelf edge (Fig. 5b), and this closely matches the suitability of environmental conditions for DSWT (Fig. 5a): the Pearson correlation coefficient between the percentage of exported kelp detritus and the suitability for DSWT is 0.92, with a *p*-value of $<0.01$.

During March, the start of peak detrital production and one of the warmest months of the year, export of simulated detritus was negligible (Fig. 5b). The horizontal density gradient during most of March was positive (water was denser moving away from the coast; Fig. S8a), and no DSWT formed (Fig. 5a). In contrast, during the remainder of peak detrital production from April to August, the horizontal density gradient was consistently negative (water was colder and denser along the coast; Fig. S8a), allowing for DSWT. However, the formation of DSWT may still be prevented by mixing of the water column due to wind^20^. This was frequently the case in April (Fig. S8b,c), and as a result, conditions were suitable for DSWT approximately 20% of the time (Fig. 5b). During the colder months of May, June, and July there were also frequent strong winds (Fig. S8c). However, these winds were often associated with onshore winter storms (coming from the west; Fig. S8d), and onshore winds enhance and strengthen the formation of DSWT^20^. As a result, DSWT occurred approximately 60% of the time in May, and peaked at $>60\%$ in June (Fig. 5b). The amount of exported simulated detritus past the continental shelf showed a corresponding increase (Fig. 5a) in these months as well. Note that although most DSWT was expected to occur in June, most simulated kelp detritus was exported in May. This is because detritus production (and correspondingly the number of particles that we released in our simulation) in May is larger than in June (black crosses in Fig. 5a). During August and September, a transition into warmer weather starts and the amount of exported detritus decreased as the conditions suitable for DSWT decreased as well.

**Figure 6:**
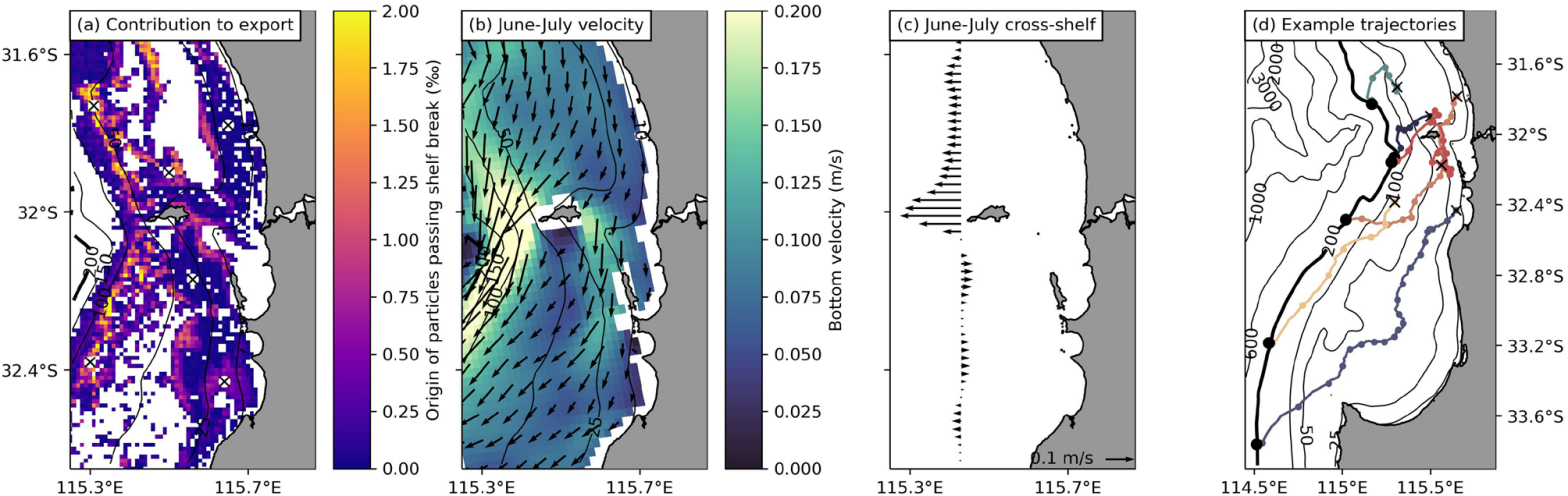
Spatial variation of the export of simulated kelp detritus, ocean bottom currents, and offshore transport. **(a)** Contribution of different kelp reefs to the export of decomposing detritus past the continental shelf edge, shown per thousand per 0.01°pixel. This figure combines information based on the amount of simulated kelp detritus released per pixel (Fig. S10b), which depends on the probability of kelp (Fig. S10a, Fig. 1a); the amount of simulated kelp detritus that is transported past the shelf edge per pixel (Fig. S10c); and, by accounting for decomposition, how long it takes simulated kelp detritus to make it past the shelf edge per pixel (Fig. S10d). Map of the June and July 2017 mean bottom velocities from the CWA-ROMS model: **(b)** spatial variation; and **(c)** cross-shelf transport component only (taken along the 100 m depth contour, and defined as being directed perpendicular to the depth contour, as in Fig. 1d). **(d)** Example trajectories of simulated kelp detritus transported past the continental shelf edge (black dot indicates where each particle crosses the shelf) released from six different locations (black crosses, also shown in panel (**a**)). The trajectories shown are those of particles that took the median time to cross the continental shelf edge of all particles that were transported past the shelf edge from that pixel (trajectories of particles that took the shortest and longest time are shown in Fig. S12a,b respectively). The dots on each trajectory are spaced 1 day apart to give an indication of how fast particles were moving in each location.

These simulation results highlight that DSWT is a key driver of the export of kelp detritus beyond the continental shelf edge, with most export happening during the colder months when this mechanism occurs most frequently, and when cross-shelf velocities along the seafloor are typically $>0.15$ m/s (Fig. 6b,c).

### Contribution from different kelp forests

So far, we considered the overall export of simulated kelp detritus and its seasonal variation. There is also great spatial variation in the amount of simulated detritus that was exported beyond the continental shelf edge. As a result, not all kelp reefs contribute equally to the overall export. The contribution of each kelp reef depends both on their vicinity to the continental shelf edge and on local oceanographic features.

Deeper kelp reefs closer to the continental shelf edge typically contributed more to the overall export of simulated kelp detritus (Fig. 6a), with almost all detritus making it past the continental shelf edge (Fig. S11). Kelp reefs north of Wadjemup also contributed more to the export than reefs to the south. This is a result of local oceanography and topography: because of the shallower region between Perth and Wadjemup, flow is generally directed more southwesterly to the north of this shallow area (Fig. 6b). As a result, the cross-shelf transport is also directed offshore to the north of and around Wadjemup and into the Perth canyon (Fig. 6c).

Although DSWT is mainly responsible for the offshore transport of simulated kelp detritus, detritus is not transported offshore in direct pathways (Fig. 6d, Fig. S12). Local oceanographic features, such as the southward flowing Leeuwin Current (Fig. 1c) as well as local mesoscale and sub-mesoscale eddies also influence the trajectories of simulated detritus. Simulated detritus from shallower kelp reefs south of Wadjemup in particular had very long and meandering trajectories before they finally crossed the continental shelf edge. Because it takes longer for simulated kelp detritus from these forests to pass the continental shelf (Fig. S10d), they contribute less to the overall export of simulated detritus (when taking decomposition into account).

### Kelp detritus carbon export estimates

Assuming 2.15 g fresh weight/kelp/day of detritus is produced (the mean yearly detrital production based on data from de Bettignies et al.^13^, Fig. 1b), an average kelp density of 6.3 kelp/m$^2$^27^, a fresh weight to dry weight ratio of 0.20^27^, a carbon content of 30% (for dry weight)^13,27^, and that 17 to 29% decomposing kelp detritus is transported past the continental shelf edge (as determined in this study): an estimated upper limit of 0.50 to 0.86 Mg C/ha/year is potentially transported past the WCS. This estimate is roughly two times larger than initial estimates per hectare for the entire Great Southern Reef^27^.

## Discussion

One of the main ways in which kelp forests can contribute to carbon sequestration is by the export of detritus to the deep sea^10^. For this to happen, cross-shelf transport must overcome the generally stronger along-shelf transport^12^ to drive detritus past the continental shelf edge before it remineralizes. Here, we showed that dense shelf water transport (DSWT) is an effective seasonal mechanism to export negatively buoyant (sinking) kelp detritus beyond the Wadjemup continental shelf (WCS). DSWT occurs most frequently on the WCS in May and June, when a band of colder, denser water forms along the coast^20^. This is also the peak time of kelp detrital production on the WCS^13^ and when most kelp detritus was transported beyond the continental shelf edge in our simulations. During warmer months, when DSWT did not occur, the export of simulated kelp detritus beyond the shelf was minimal. This highlights the importance of DSWT as a mechanism driving cross-shelf transport of negatively buoyant kelp detritus towards the deep sea.

In addition to demonstrating DSWT as a mechanism to export kelp detritus from the WCS, our simulations also highlighted that not all reefs contributed equally to the overall export. In general, kelp reefs closer to the continental shelf edge and to the north of Wadjemup contributed more to detritus export than shallower reefs closer to shore. This illustrates the importance of both local oceanographic features, and the proximity of kelp reefs to the shelf edge, to the transport of detritus towards the deeper ocean, even on local scales.

Overall for the WCS region, we found that 51% per year of simulated kelp detritus was transported past the continental shelf edge at 200 m depth, or 17 to 29% per year when accounting for decomposition while in transit on the shelf. Here, we assumed a constant exponential decay rate across the shelf based on decomposition experiments by Simpkins et al.^25^, which were performed on the WCS up to 50 m depth. However, since we consider transport to depths $>50$ m, it is possible that decay rates will vary as detritus is transported in cooler water^28^ towards the shelf edge. To simulate the transport of kelp detritus, we used a high-resolution ocean model to force particle tracking simulations. However, as is typical for local and regional ocean models, it does not account for ocean dynamics on the scale of a reef. While this is not expected to affect the transport pathways of simulated detritus past the continental shelf, it may influence the retention time of detritus within the reef itself^29^. Depending on the reef rugosity, kelp detritus may also be captured within the reef system. This effect is not captured in our simulations. We also do not consider burial of kelp detritus in our simulations. Since there is only a very thin layer of sediment ($<1$ m) above hard substrate on the WCS^30^, this effect is of secondary importance in this region. In our simulations, particles drift passively with ocean bottom currents, which was justified by sensitivity analyses accounting for threshold velocities of small and medium-sized kelp detritus (see “Methods” section). However, large detritus may be transported differently. To account for the importance of this in particle tracking simulations, it is necessary to both understand how larger detritus is affected by different flow conditions, as well as the typical size distribution of kelp detritus. In our simulations, we assume that kelp detritus drifts with ocean bottom currents and do not explicitly account for processes related to resuspension and sinking speeds of detritus. However, since the predominant kelp species on the WCS is negatively buoyant (golden kelp—*Ecklonia radiata*), it is unlikely to resuspend above the ocean model bottom layer. Inside that framework, we expect that including resuspension and sinking effects would have a minimal effect on our simulation results. We ran simulations for 2017, as this can be considered a representative neutral year in the WCS region (neutral El Niño Southern Oscillation^31^ and Indian Ocean Dipole^32^ conditions). In future studies, we will investigate the influence of climate variability on the occurrence of DSWT and associated export of detritus.

In this study, we mainly focused on the export of simulated kelp detritus beyond the continental shelf edge at 200 m depth. This is below the depth of the Leeuwin Current^15^ as well as the typical mixed layer depth in the region^33,34^. Kelp detritus transported past the shelf edge can therefore potentially be sequestered as there is little exchange with surface waters below 200 m. However, sequestration time scales vary widely depending both on the depth and the geographic location^35^, and biogeochemical models combined with ocean circulation models are required to determine potential sequestration timescales of tens to hundreds of years^36^.

Although we focused only on the export of particulate organic carbon (detritus) in this study, the export of dissolved organic carbon (DOC) into the deep sea is also an important pathway of kelp carbon sequestration^10^. The dominant species in our study region (golden kelp—*Ecklonia radiata*) is a large producer of DOC^37^. To determine the contribution of DOC to the potential sequestration of kelp carbon, a better understanding of the decomposition time of DOC and its role in coastal biogeochemistry is needed.

The 17 to 29% export of negatively buoyant kelp detritus past the continental shelf edge estimated here, is roughly twice initial global estimates of 11%^10^ and local estimates that 4–9% of kelp detritus becomes buried in sediment at 48 m depth in Plymouth Sound (United Kingdom)^38^. Smale et al.^39^ found that $>90\%$ of negatively buoyant kelp detritus in an area with intense wave action was removed from its source population, but they did not determine its ultimate fate. In this study, we identified DSWT as the main mechanism responsible for the offshore transport of negatively buoyant kelp detritus from the coastal zone. Other studies that have considered the transport of kelp detritus from the coastal to deeper water have focused on positively buoyant (floating) kelp species, which are transported by different mechanisms. For example, Queirós et al.^40^ used particle tracking simulations to show how the export of positively buoyant kelp detritus from the coastal zone depends heavily on winds. Using particle tracking simulations for kelp farms in northern Norway, Broch et al.^41^ found that the depth that kelp detritus reached depended on both the location of the kelp farm and the detritus’ sinking speed. Kokubu et al.^42^ confirmed the presence of positively buoyant *Sargassum horneri* originating from the northeastern coast of Japan up to 800 km offshore and in water depths up to 1800 m and related this to the presence of ocean fronts. In contrast, Ager et al.^43^ found that 80% of positively buoyant kelp detritus was retained within a Greenland fjord and was not transported into the open ocean. These studies highlight the need to consider different transport mechanisms for kelp species with different buoyancy, as well as the relevant dynamics in different regions.

The relatively high export efficiency of kelp detritus past the continental shelf edge at 200 m depth that we find here, is a result of DSWT. DSWT occurs in many mid-latitude locations around the world^17^, which is also where kelp forests are most productive^8^. Since DSWT is an effective mechanism for the offshore transport of negatively buoyant kelp detritus, the global export of kelp detritus may be larger than previously estimated.

Our estimated export percentages are also of the same order, or exceed, global estimates of the export efficiency of phytoplankton from the ocean surface layer through the biological gravitational pump, which vary between 7 and 26%^44^. This may indicate that the lateral transport of carbon from the coast to the deep ocean may be more important than previously acknowledged, and may require revisiting the mechanisms that contribute to the biological carbon pump.

## Methods

To simulate the transport of kelp detritus on the WCS, we use Lagrangian particle tracking simulations forced by bottom ocean currents from a regional ocean model, CWA-ROMS. The following sections describe in more detail the characteristics of CWA-ROMS; the kelp probability map that we use to determine release locations of particles; the particle tracking simulations themselves; and finally the method that we used to determine suitable conditions for DSWT to form (used in Fig. 5b).

### Ocean circulation model (CWA-ROMS)

CWA-ROMS is a high-resolution realistic set-up of the Regional Ocean Modelling System (ROMS)^45^ of the central west coast of Australia (CWA). CWA-ROMS was run in reanalysis mode from 2000 until 2022. Ocean boundary and initial conditions were provided by the operational analysis and forecast system of Mercator Ocean International with 1/12° horizontal resolution. Atmospheric forcing was provided by the European Centre for Medium-Range Weather Forecasts (ECMWF) ERA-5 model output at 25 km horizontal resolution and hourly temporal resolution. Tidal forcing was provided by the TPXO9 global tidal solution^46^. Mahjabin et al.^24^ validated that the model nested in CWA-ROMS accurately reproduced DSWT along the WCS by comparing simulation results with DSWT events measured by ocean gliders.

In this study, we ran particle tracking simulations for 2017. We chose 2017 because this was a neutral El Niño^31^ and neutral/slightly positive Indian Ocean Dipole year^32^ (Fig. S2). Both these climate modes influence variability in Western Australia, so 2017 can be considered a representative neutral year.

CWA-ROMS returns output every 3 h. The horizontal resolution of CWA-ROMS in the region that we are interested in is approximately 2 km, but varies slightly between 1.7 and 2.1 km because it uses a curvilinear grid (Fig. S3a). CWA-ROMS has 25 vertical sigma (terrain-following) layers, which means that the thickness of the bottom layer varies depending on the overall depth of the water column. In the region that we are interested in, the bottom layer thickness varies between $<1$ m up to 260 m (Fig. S3b). On the continental shelf, the bottom layer thickness is typically between $<1$ and 10 m. Because the predominant kelp species on the WCS is negatively buoyant, we only used ocean currents from the bottom layer of CWA-ROMS to force particle tracking simulations.

### Kelp probability map

To determine the release locations of particles in our particle tracking simulations, we used a kelp probability map which was developed using species distribution models (SDMs) following methods outlined in Hovey et al.^47^ and applied to the full extent of kelp in Bellchambers et al.^48^ and Giraldo-Ospina^49^. In brief, a species distribution model was developed for kelp using the national $250 \times 250$ m bathymetry and topography grid^14^, derived terrain variables as predictor variables, and a collation of geo-referenced benthic habitat data which contained kelp presence and absence. The modeling output was a spatially explicit map showing probability of kelp occurrence with probabilities close to zero indicating areas where kelp is highly unlikely to be present and close to one indicating areas where kelp is highly likely to be present.

### Particle tracking simulations

We used OpenDrift v1.10.5^50^ to run particle tracking simulations forced by 2017 bottom ocean currents from CWA-ROMS. We used a 4th order Runge–Kutta advection scheme and included random Brownian motion with a constant horizontal diffusivity of $K_h = 10$ m/s$^{2}$, based on the equation suggested by Peliz et al.^51^.

We released particles daily from the 1st of January until the 31st of December and ran the simulation until the end of January 2018, to allow the particles released in December to be transported. We randomly released particles depending on the kelp probability; effectively releasing more particles in areas with a higher probability. We then scaled the number of particles released each day by the detrital production estimated by de Bettignies et al.^13^ (Fig. 1b). Overall, we released a total of 127,906 particles.

Particles were passively transported by bottom layer ocean currents in our simulations. However, because the bottom layer thickness varies depending on the overall depth of the water column (Fig. S3b), the bottom layer currents represent ocean currents at different heights above the seafloor. It is possible to correct for this, by assuming a logarithmic bottom velocity profile (Fig. S4); this was for example done in kelp detritus transport simulations by Broch et al.^41^. We ran particle tracking simulations for the peak detrital period from March until August including this correction and found that the sensitivity of our simulation results to this correction was minimal (Fig. S5). We therefore did not apply this correction in our simulation.

In addition, negatively buoyant kelp detritus has a threshold velocity: a minimum velocity that ocean currents need to reach before detritus will start to move along the seafloor and be transported by ocean currents. Filbee–Dexter (unpublished data) found in flume experiments that the threshold velocity for *Ecklonia radiata* was $0.045 \pm 0.016$ m/s for small pieces of detritus and $0.031 \pm 0.015$ m/s for medium sized pieces. The more conservative threshold velocity of 0.045 m/s was exceeded 54% of the time by bottom CWA-ROMS currents during our simulation time (Fig. S6a), but this percentage varied widely depending on the location and was exceeded $>\,80\%$ of the time on the continental shelf (Fig. S6b). We ran particle tracking simulations for the peak detrital period from March until August accounting for the threshold velocity of 0.045 m/s and found that the sensitivity of our simulation results when including the threshold velocity was minimal (Fig. S7). We therefore did not apply a threshold velocity but instead let particles drift passively with bottom layer ocean currents.

We applied decomposition to particles as a processing step after the particle tracking simulation had been run. The remaining fraction of a particle *P* in time was calculated as

1 $$\begin{aligned} P(t) = P_0 e^{kt}, \end{aligned}$$

where $P_0 = 1.0$ is the initial particle fraction, *t* is the age of a particle after it has been released in days, and $k = - \,0.075 \pm 0.031$ per day is the decay rate for *Ecklonia radiata* as determined in situ on the WCS by Simpkins et al.^25^.

### Suitable conditions for dense shelf water transport

DSWT is a gravitational circulation that requires a negative horizontal density gradient (increasing density towards the coast) to form. This can occur if water along the coast becomes more saline (which, for example, happens in the hypersaline Shark Bay^52^), colder, or a combination of both, than water further offshore. On the WCS the main driver of negative density gradients is the temperature, and DSWT forms as a result of differential cooling during the colder months^20^. In addition to a negative horizontal density gradient, the water column also needs to be stratified for DSWT to occur. Mixing due to tides and wind can prevent the formation of DSWT^52^. The WCS is a microtidal region, with a tidal range of only $\sim$ 0.5 m, so mixing due to tides is negligible^20^. Mixing due to winds can be significant on the WCS: during the warmer months, Western Australia experiences some of the strongest seabreezes in the world^22,23^, and in colder months experiences frequent storms. Although high wind speeds in general tend to destratify the water column, onshore winds (especially associated with cold fronts) can enhance the negative density gradient and promote and strengthen the formation of DSWT^20^.

To determine when environmental conditions are suitable for DSWT, we combined the following requirements. First, there needs to be a negative horizontal density gradient present along the coast: $\frac{\partial \rho }{\partial x} < 0$ (Fig. S8a).

Second, the water column needs to be stratified. This can be determined using the potential energy anomaly (PEA), $\phi$, defined as^53^:

2 $$\begin{aligned} \phi = \frac{1}{h}\int \limits_{-h}^0(\bar{\rho }-\rho )gzdz, \end{aligned}$$

where *h* is the water depth, $\rho$ is the water density, $\bar{\rho } = \frac{1}{h}\int _{-h}^0\rho dz$ is the vertically averaged water density, and $g = 9.81$ m/s$^{2}$ the gravitational acceleration. PEA (in J/m$^{3}$) is a measure for the amount of energy per unit volume required to fully mix the water column. Generally, $\phi = 0$ indicates that the water column is fully mixed. However, because in this analysis we average $\phi$ over a larger region, we find that the condition $\phi > 5$ applies best to the WCS. Figure S9 shows examples of both a fully mixed water column (with an average $\phi = 3.5$) and a stratified water column (with an average $\phi = 11.9$). It also shows maps of how $\phi$ varies spatially in these two examples. So, our second condition is that $\phi > 5$ (Fig. S8b).

Third, the stratifying influence of the gravitational circulation needs to be larger than the destratifying influence of wind mixing^20,52^:

3 $$\begin{aligned} \underbrace{\frac{1}{320}\frac{g^2h^4}{\rho K_{mz}}\left( \frac{\partial \rho }{\partial x}\right) ^2}_\text {gravitational} > \underbrace{\delta \kappa _s \rho _a \frac{W^3}{h}}_\text {wind mixing}, \end{aligned}$$

where, following Hetzel et al.^52^, $K_{mz} = 1 \times 10^{-2} \text { m}^2/\text {s}$ is the vertical eddy diffusivity, $\delta =3 \times 10^{-3}$ is the wind mixing efficiency, $\rho _a=1.2 \text { kg}/\text {m}^{3}$ is the density of air, *W* is the wind speed, and $\kappa _s = \frac{0.03(0.63+0.066 W^{1/3})}{1000}$ is the drag coefficient for surface wind stress. Our third condition is that Eq. (3) must hold (Figure S8c), except if the wind is directed onshore (defined for the WCS as between 225 and 315°: between northwesterly and southwesterly wind; Fig. S8d).

If all the above three conditions hold (Fig. S8e), then we consider conditions suitable for DSWT to form.

### Supplementary Information

Supplementary Figures.

## Data Availability

Satellite sea surface temperatures and ocean glider data were sourced from Australia’s Integrated Marine Observing System (IMOS)—IMOS is enabled by the National Collaborative Research Infrastructure Strategy (NCRIS). All ocean gliders were operated by the ocean glider facility at the University of Western Australia. CWA-ROMS data is freely available and can be requested through the corresponding author MvdM. The code to run particle tracking simulations and for all the analyses is available on: https://github.com/mheen/kelp_export_wcs. Particle tracking simulation results can also be requested from the corresponding author MvdM.
