# Supplementary material for: Substantial kelp detritus exported beyond the continental shelf by dense shelf water transport
Source: Sci Rep. 2024 Jan 8;14:839. doi: 10.1038/s41598-023-51003-5 (PMC10774291; doi:10.1038/s41598-023-51003-5)
Supplement: Supplementary file 1 — Supplementary Figures. [file 41598_2023_51003_MOESM1_ESM.pdf]

Supporting Material for

Substantial kelp detritus exported beyond the continental shelf by dense shelf water transport

Mirjam van der Mheen[1], Thomas Wernberg[1,2], Charitha Pattiaratchi [3], Albert Pessarrodona[1], Ivica Janekovic[3], Taylor Simpkins[1], Renae Hovey[1], Karen Filbee-Dexter[1,2]

[1]School of Biological Sciences and UWA Oceans Institute, University of Western Australia, Perth, WA, Australia
[2]Institute of Marine Research, Nye Flødevigveien 20, His, 4817 Norway
[3]Oceans Graduate School and UWA Oceans Institute, University of Western Australia, Perth, WA, Australia

This supporting information provides additional figures:

Supporting Methods

S1 An example illustrating that our numerical ocean model CWA-ROMS simulates dense shelf water transport.

S2 El Niño and Indian Ocean Dipole climate indices illustrating that 2017 can be considered a representative neutral year on the Wadjemup continental shelf (WCS).

S3 Maps showing the horizontal and vertical resolution of the CWA-ROMS model on the WCS.

S4 Logarithmic velocity profile corrections needed to adjust CWA-ROMS ocean bottom currents to 0.5 m above the seafloor on the WCS.

S5 Sensitivity analyses indicating that including the logarithmic velocity profile corrections (Figure S4) has a negligible effect on particle tracking simulation results.

S6 Exceedance of threshold velocities for small *Ecklonia radiata* detritus by CWA-ROMS ocean bottom currents on the WCS.

S7 Sensitivity analyses indicating that including the threshold velocity (Figure S6) has a negligible effect on particle tracking simulation results.

Figures S4 to S7 justify the use of passively drifting particles transported by ocean bottom currents in our particle tracking simulations.

S8 Timeseries of different requirements for suitable conditions for dense shelf water transport to form (used to determine Figure 5b in the main manuscript).

S9 Examples showing a fully mixed and stratified water column and the corresponding potential energy anomaly ϕ, to illustrate the use of the requirement $\phi > 5$ in Figure S8b.

Supporting Results

S10 Maps illustrating the components that determine the contribution of different kelp reefs to the export of simulated kelp detritus past the continental shelf edge (Figure 6a in the main manuscript).

S11 Map illustrating the export efficiency of simulated kelp detritus transported past the continental shelf edge. Where Figure 6a in the main manuscript shows how each reef contributes to the total successfully exported detritus, this figure shows the percentage of detritus that is successfully exported from each initial location.

S12 Example trajectories of simulated kelp detritus transported past the continental shelf edge. Figure 6d in the main manuscript shows the trajectories of particles taking the median time to make it past the shelf edge from different locations; this figure shows the trajectories of particles taking the minimum and maximum time.

Supporting Methods

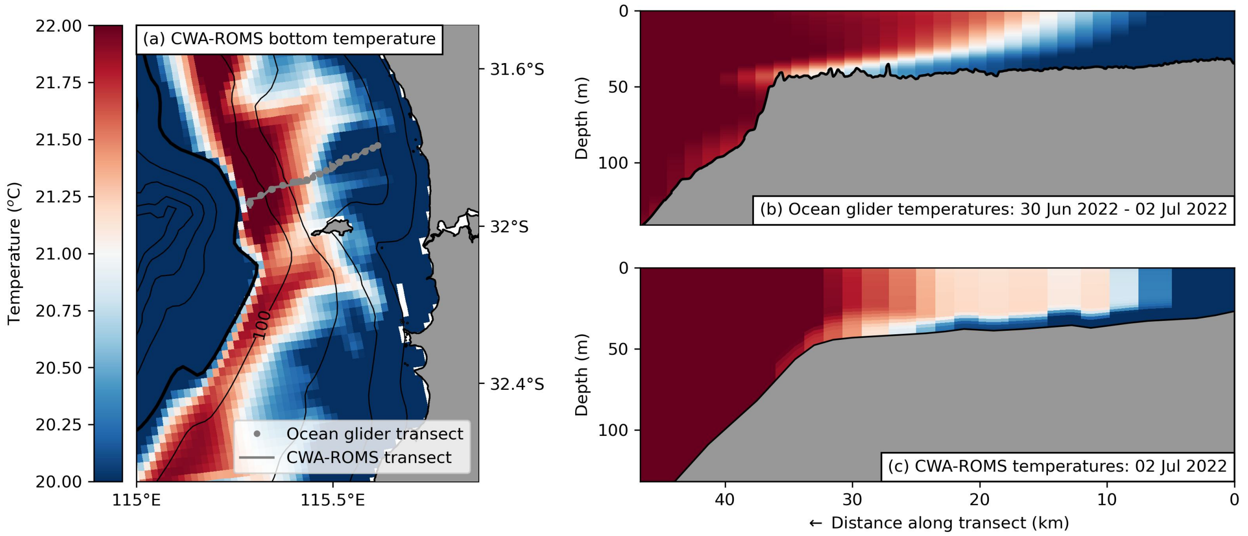

Figure S1 **(a)** Map of CWA-ROMS daily mean bottom temperature on the 2nd of July 2022. **(b)** Ocean glider temperature measurements from IMOS along a transect (shown with grey dots in (a)) during a mission from the 30th of June until the 2nd of July 2022 (also shown in Figure 2b in the main manuscript). **(c)** CWA-ROMS daily mean temperature on the 2nd of July 2022 along the same transect as the glider path (shown with a grey line in (a)). CWA-ROMS simulates the occurrence of dense shelf water transport.

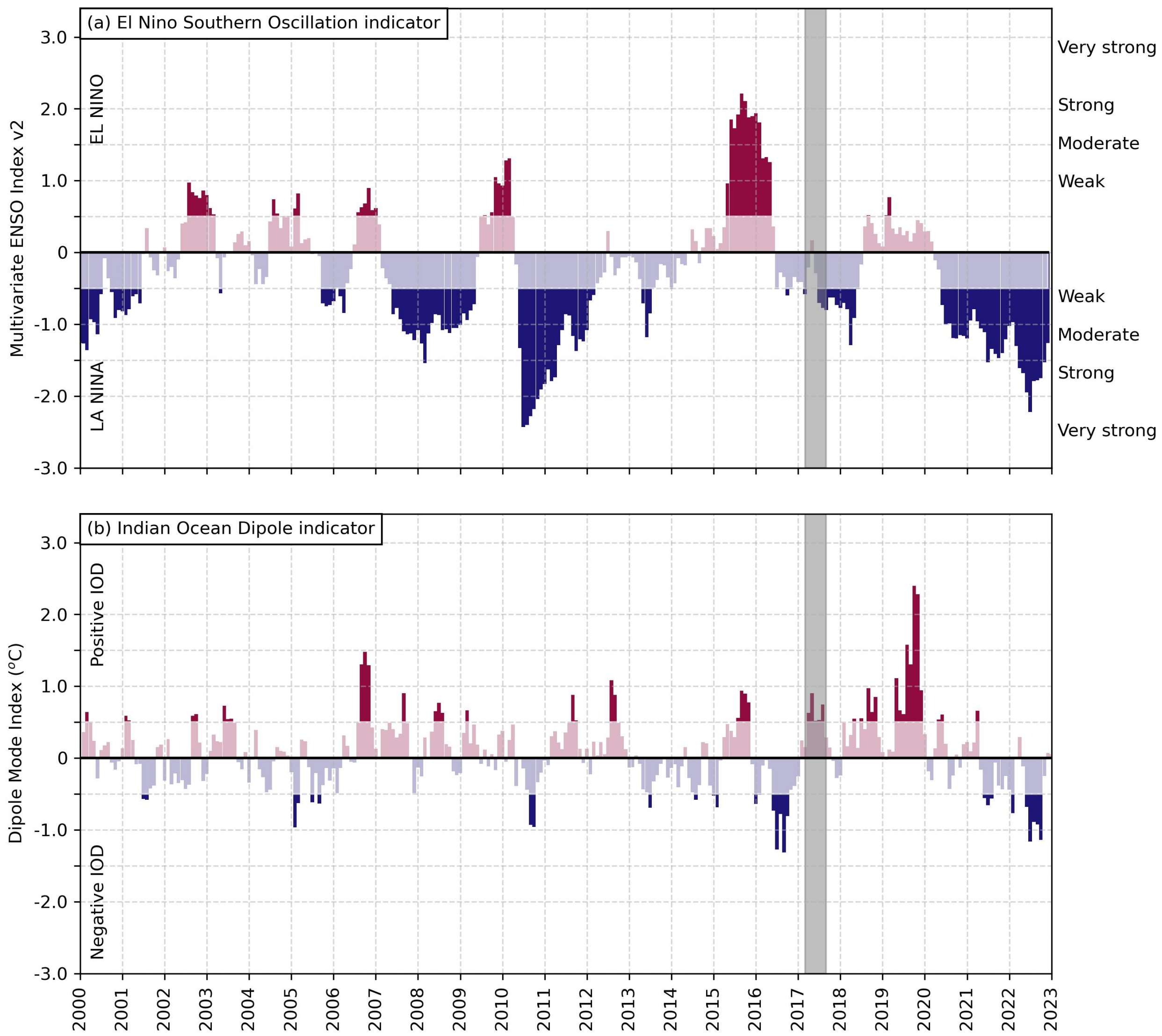

Figure S2 Relevant climate indices for Western Australia: **(a)** El Niño Southern Oscillation (ENSO) index; and **(b)** the Indian Ocean Dipole (IOD) mode index, from 2000 until 2022. The grey bar in both panels shows the period for which we simulate the transport of kelp detritus on the Wadjemup continental shelf. We chose 2017 because this was a neutral ENSO year, combined with a mild IOD.

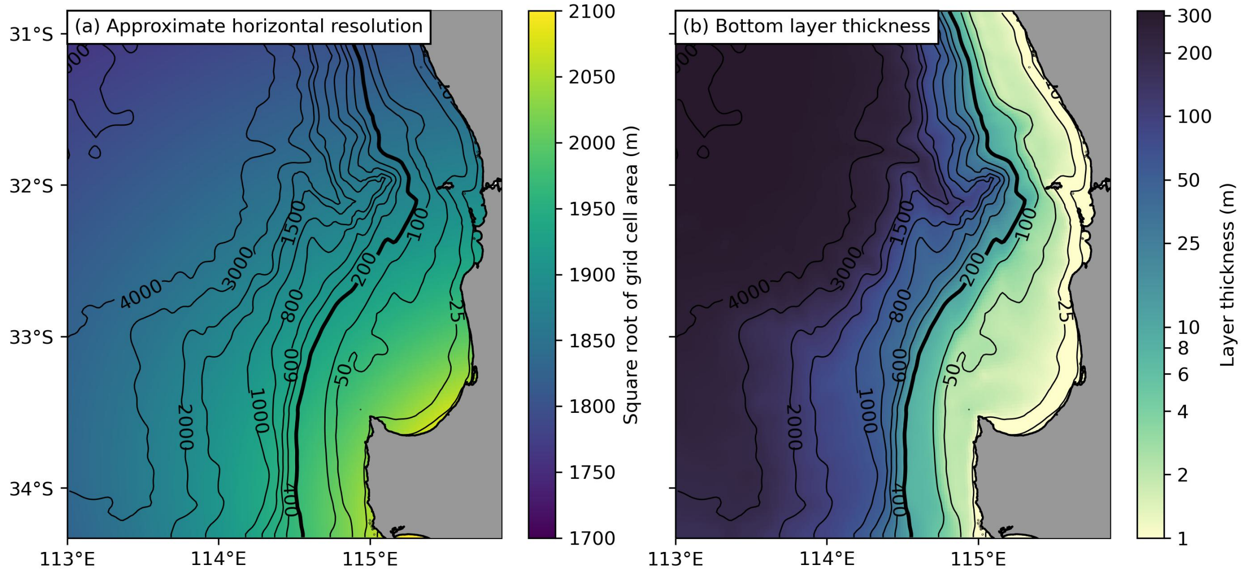

Figure S3 **(a)** Horizontal resolution of the CWA-ROMS model that we use to force our particle tracking simulations with. This model has a curvilinear grid, as a result, the horizontal resolution varies slightly between different locations. **(b)** Thickness of the CWA-ROMS model bottom layer. This model uses a terrain-following sigma-coordinate system, which means that the thickness of each model layer varies depending on the depth. On the continental shelf, the thickness of the bottom layer varies between <1 and 10 m. In the deeper ocean, the thickness can reach 250 m or more.

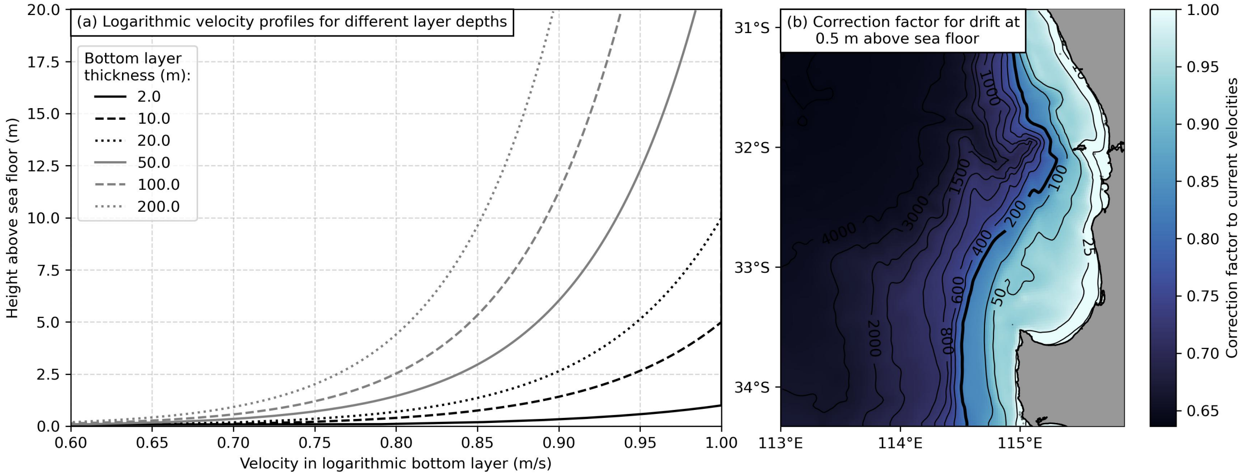

Figure S4 Because the thickness of the bottom layer in the CWA-ROMS model varies per location (Figure S3b) the modeled ocean currents in the bottom layer represent currents at different heights above the sea floor. It is common practice to assume that the speed of ocean currents varies logarithmically between the sea floor and the model output in the bottom layer (this same approach is used in ROMS when ocean current velocities are calculated). Panel **(a)** illustrates what the logarithmic velocity profiles look like for different bottom layer thicknesses. Assuming that kelp detritus drifts roughly 0.5 m above the sea floor, this means that in deeper water (thicker bottom layer) the velocity with which kelp detritus drifts is overestimated if we use the modeled bottom layer ocean currents. Using the logarithmic profile assumption, we can correct for this. Panel **(b)** shows the correction factor that would be needed to get the ocean current velocities at 0.5 m above the sea floor. On the continental shelf, the bottom layer velocities would need to be multiplied by a factor between 1.0 (no correction) and approximately 0.90 (reduced by 10%). In deeper water, the correction factor drops down to a minimum of approximately 0.65 (reduction of 35%).

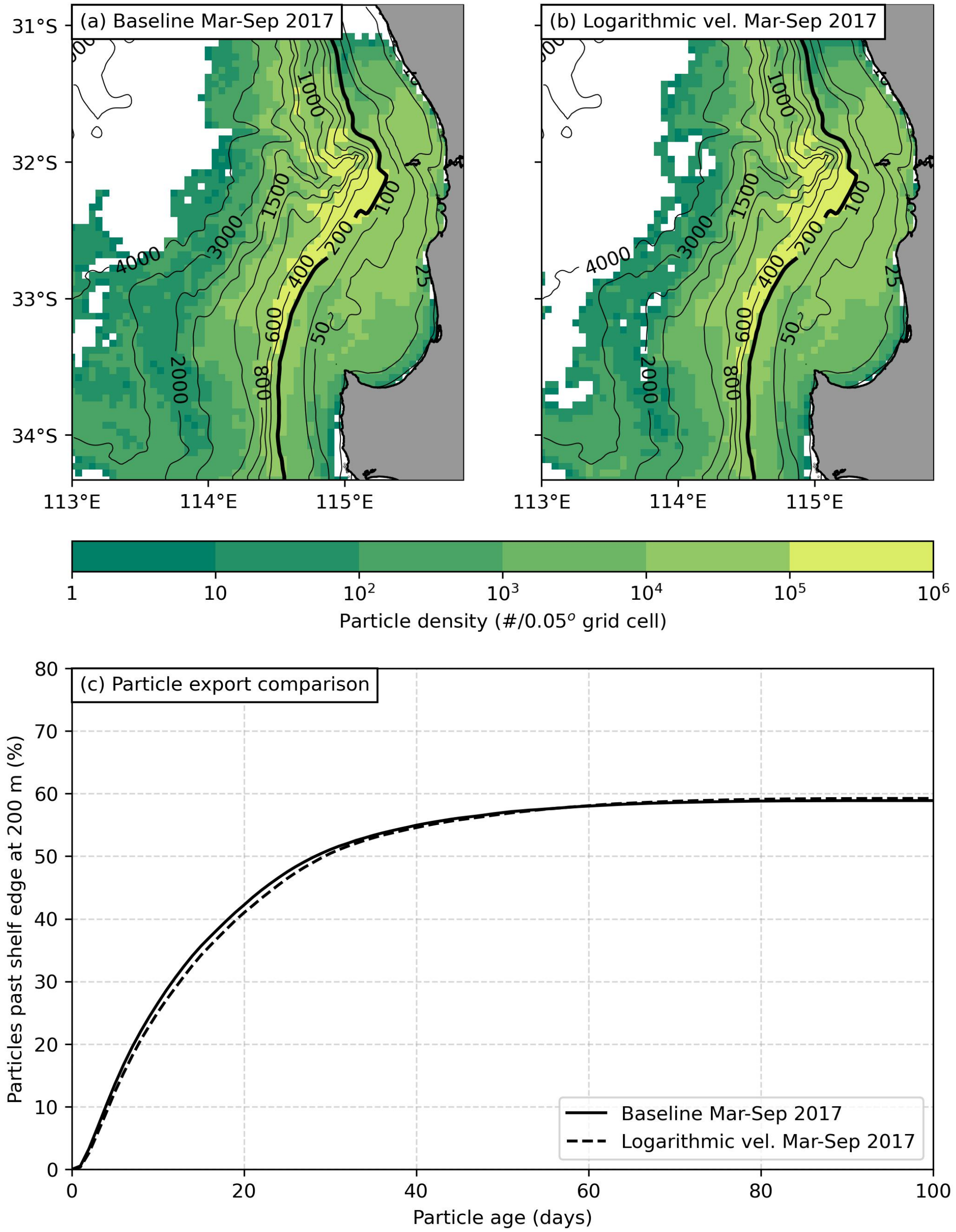

Figure S5 To simulate the transport of kelp detritus drifting just above the sea floor (we assume 0.5 m above the sea floor), the bottom layer ocean currents from the CWA-ROMS model need to be corrected (Figure S4). To determine the sensitivity of our simulation results to this logarithmic profile correction, we compared particle tracking simulation results with and without this correction. We show here that the sensitivity of the simulation results to this correction is minimal. The general patterns of the total particle density from March until September (with particles released from March until August; the peak detrital production period) without correction **(a)** and with correction **(b)** are almost identical, except at depths exceeding 2000 m (where the correction factors are strongest, see Figure S4b). Since we are interested in the transport of simulated kelp detritus on the continental shelf and just off the continental shelf edge, the sensitivity is negligible. The percentage of simulated kelp detritus that is exported beyond the continental shelf edge **(c)** shows a negligible sensitivity to the logarithmic bottom layer correction as well.

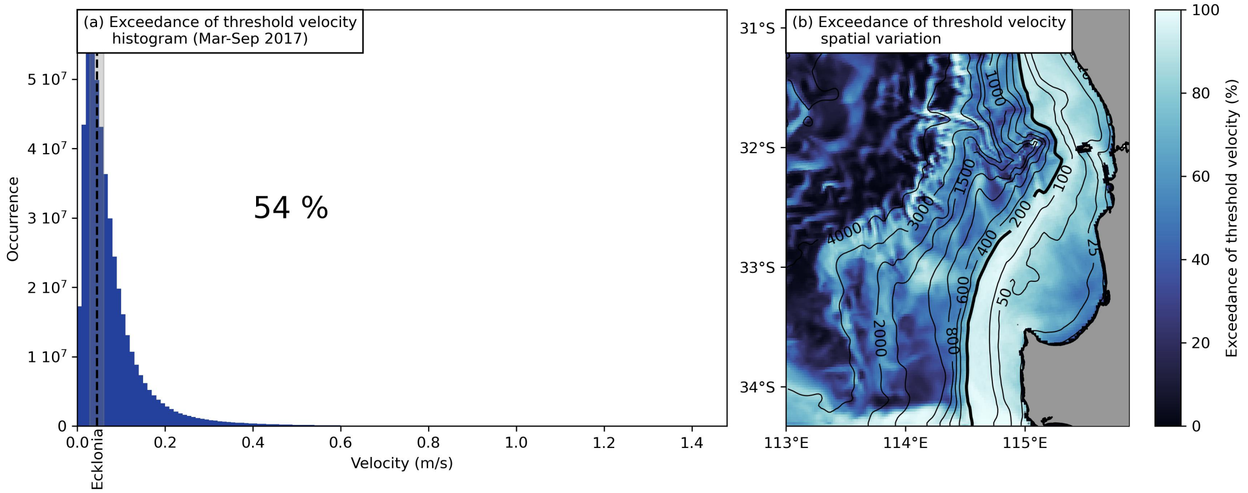

Figure S6 The threshold velocity (the velocity after which negatively buoyant objects start to move with ocean currents) for different sizes of *Ecklonia radiata* detritus was measured in flume experiments by Filbee-Dexter et al. (unpublished data). They found a threshold velocity of 0.045 ± 0.016 m/s for small pieces of detritus and of 0.031 ± 0.015 m/s for medium sized pieces. We use the threshold velocity of small pieces here, since it is the more conservative estimate (higher threshold velocity). Panel **(a)** shows the occurrence of bottom layer ocean velocities from the ROMS-CWA model from March to September 2017. The dashed black line indicates the mean threshold velocity of 0.045 m/s, with the grey band showing the mean $\pm$ standard deviation. Bottom layer ocean currents exceeded the threshold velocity of 0.045 m/s 54% of the time. However, the exceedance of the threshold velocity varies strongly per location, as shown in panel **(b)**. On the continental shelf, the threshold velocity was exceeded between 80-100% of the time, while in the deeper ocean >4000 m depth it was rarely exceeded.

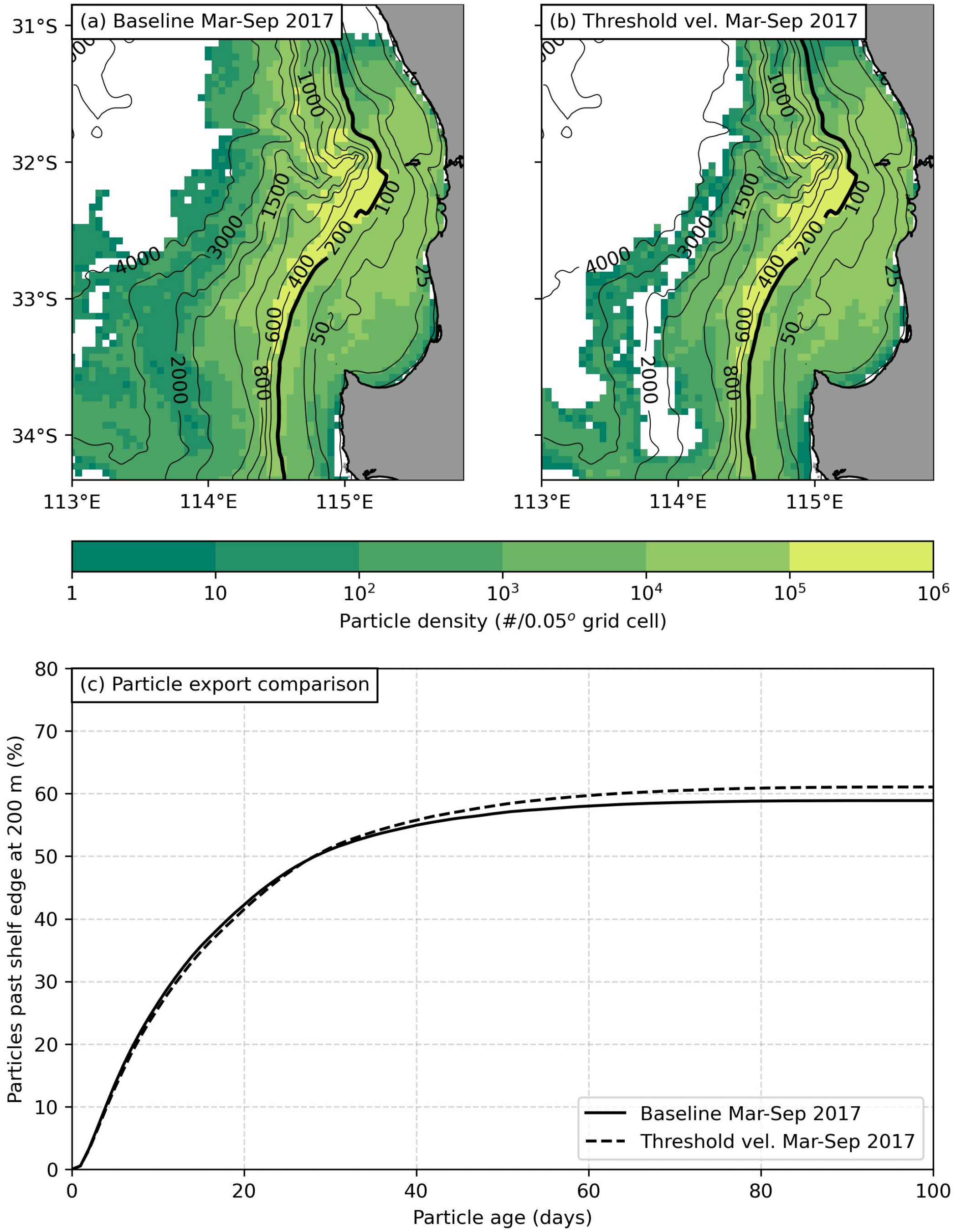

Figure S7 To simulate the transport of kelp detritus drifting along the sea floor, its threshold velocity (Figure S6) needs to be considered. In particle tracking simulations, the threshold velocity is taken into account by only allowing particles to be transported by ocean currents if their velocity exceeds the threshold velocity. To determine the sensitivity of our simulation results to a threshold velocity of 0.045 m/s, we compared particle tracking simulation results with and without a threshold velocity. We show here that the sensitivity of the simulation results to this threshold velocity is minimal. The general patterns of the total particle density from March until September (with particles released from March until August; the peak detrital production period) without threshold velocity **(a)** and with threshold velocity **(b)** are almost identical, except at depths exceeding approximately 1500 m. Since we are interested in the transport of simulated kelp detritus on the continental shelf and just beyond the continental shelf edge, the sensitivity is negligible for our purposes. The percentage of simulated kelp detritus that is exported beyond the continental shelf edge **(c)** also shows a negligible sensitivity to the inclusion of a threshold velocity.

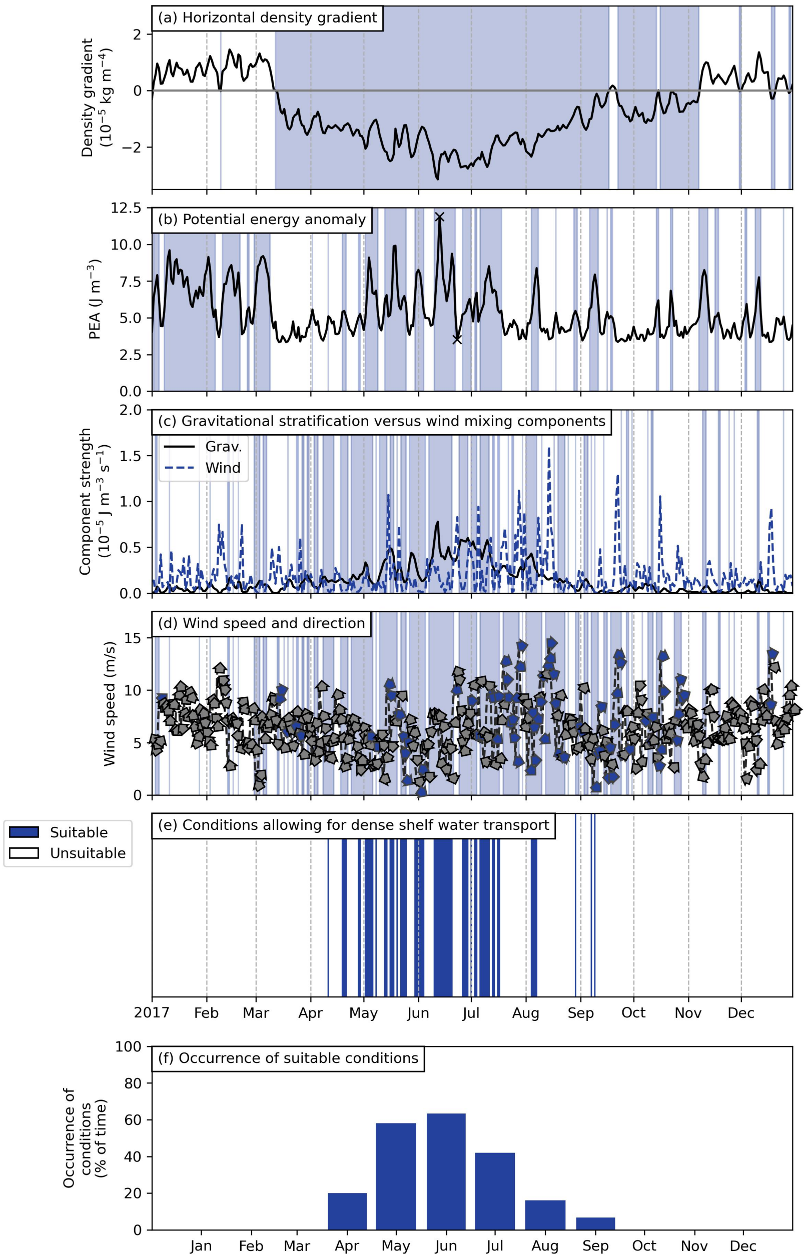

Figure S8 Timeseries averaged over the Perth region of the Wadjemup continental shelf using CWA-ROMS ocean model data and ECMWF ERA5 wind model data, of: **(a)** horizontal density gradient; **(b)** potential energy anomaly (PEA: a measure for the stratification of the water column, for increasingly positive PEA the water column is more stratified); **(c)** the strength of opposing components in the formation of dense shelf water transport: gravitational stratification versus wind mixing; **(d)** wind speed (dashed black line) and direction (arrows); **(e)** the overall suitability of environmental conditions for dense shelf water transport. We determine the overall suitability using several requirements: 1) the horizontal density gradient is negative, $\frac{\partial\rho}{\partial x} < 0$, indicating that there is higher density water near the coast; 2) the potential energy anomaly is larger than 5.0 J/m^3, $\phi > 5$, indicating that the water column is stratified; 3) the stratifying influence of the gravitational circulation, $\frac{1}{320}\frac{g^2h^4}{\rho K_{mz}}\left(\frac{\partial\rho}{\partial x}\right)$, is larger than the destratifying influence of wind mixing, $\delta\kappa_S\rho_a\frac{W^3}{h}$, unless the wind is directed onshore (defined here as between 225° and 315°). The light blue shaded areas in each panel indicate when the requirements for each of these conditions have been met. **(f)** The percentage of time for each month that conditions are suitable for dense shelf water transport.

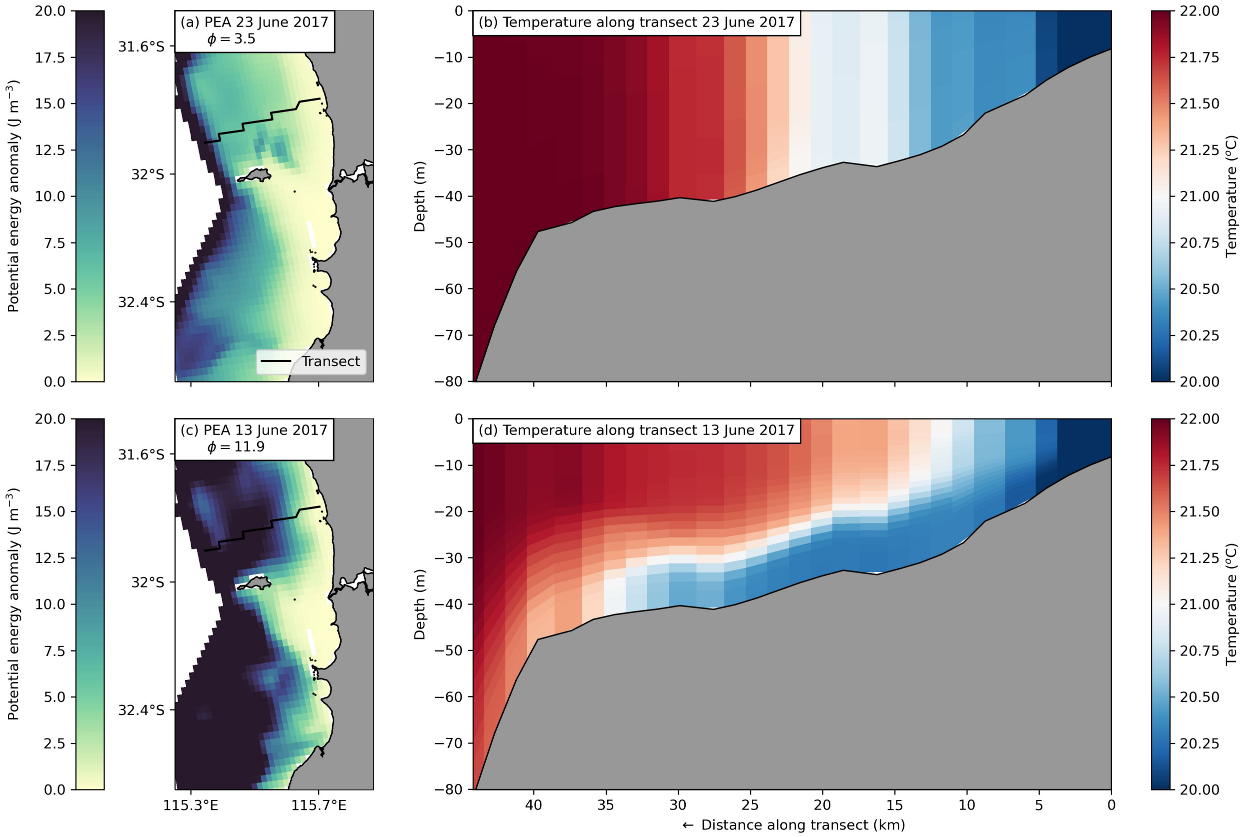

Figure S9 Maps of potential energy anomaly (PEA, ϕ) for a day in June 2017 where the overall mean PEA was relatively: **(a)** low, at $\phi = 3.5$ J/m^3; and **(c)** high, at $\phi = 11.9$ J/m^3 (indicated by black crosses in Figure S8b). Corresponding ocean temperatures throughout the water column along the transect shown with a black line in panel (a) and (c) for a relatively: **(b)** low PEA, showing a fully mixed water column; and **(d)** high PEA, showing a stratified water column with, in this case, a dense shelf water transport event occurring. These are typical examples, and we find that for $\phi \approx 5$ the water column changes from mixed to stratified, which is why we used the condition of $\phi > 5$ to determine suitable conditions for dense shelf transport events (Figure S8).

Supporting Results

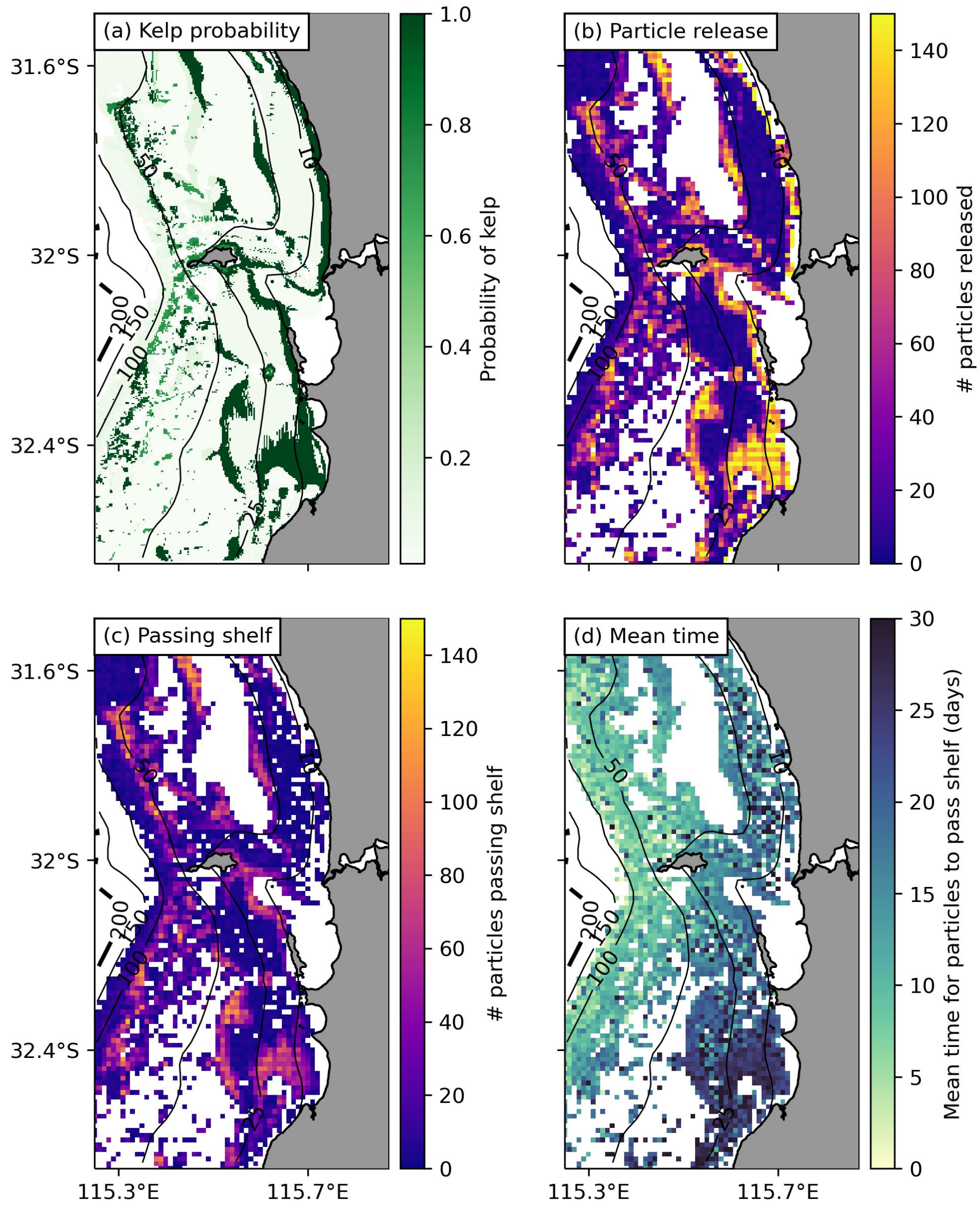

Figure S10 **(a)** Probability of kelp in the Perth region on the Wadjemup continental shelf. **(b)** The number of particles released per pixel. The number of particles released per location depends on the kelp probability shown in panel (a): the higher the probability of kelp, the more particles are released. **(c)** The number of particles that are exported past the continental shelf edge from each pixel, which depends both on local oceanographic features, the proximity of a pixel to the continental shelf edge, and the number of particles released from each pixel. **(d)** The mean time that it takes particles to be exported past the continental shelf edge from each pixel, which determines how far simulated detritus is decomposed. This mean time mainly depends on local oceanographic features and the proximity to the continental shelf edge of each pixel. The overall contribution of each pixel to the total export of simulated kelp detritus past the continental shelf edge (Figure 6a in the main manuscript) is a combination of all these factors.

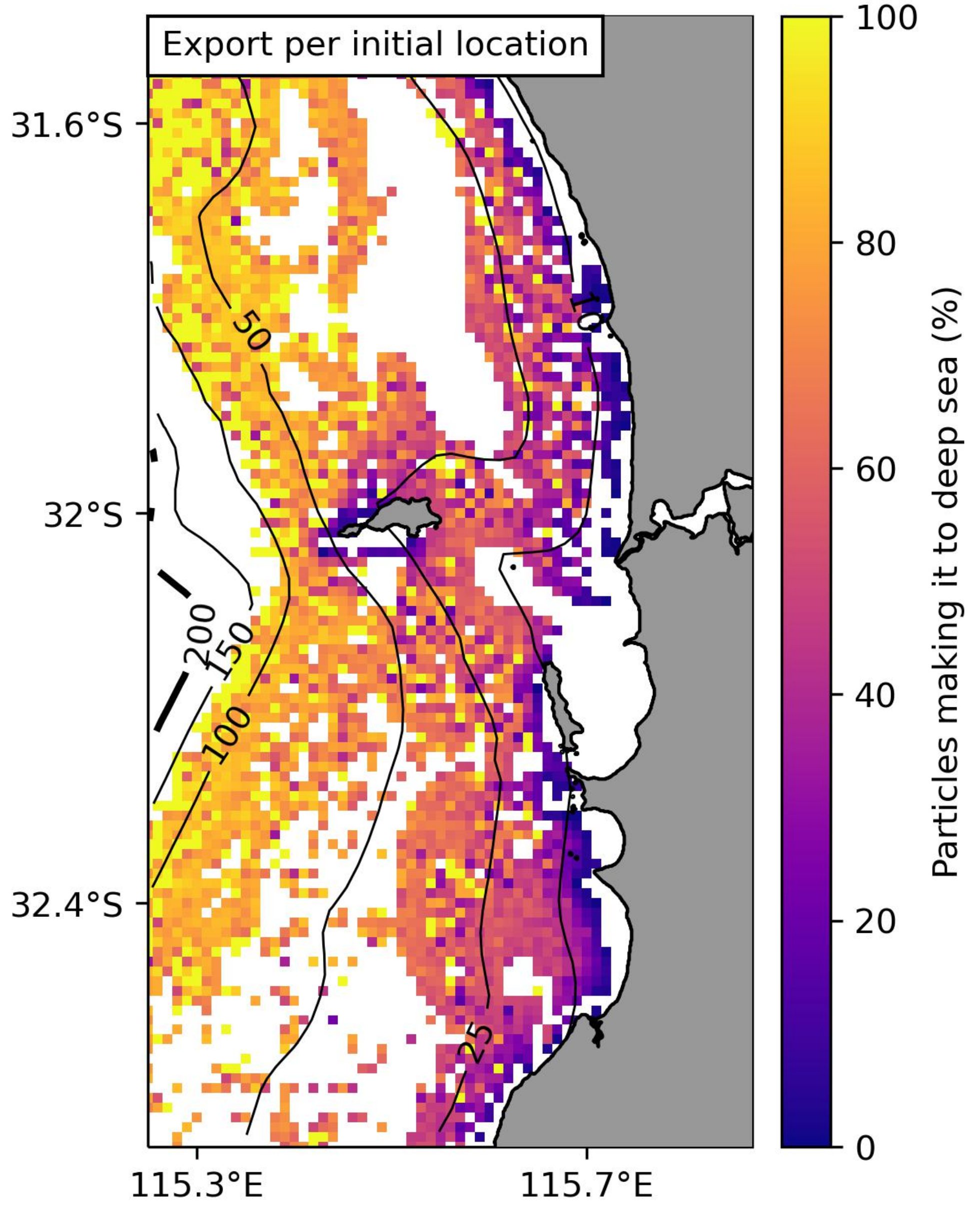

Figure S11 The percentage of simulated kelp detritus that is exported past the continental shelf edge from each pixel over a full year.

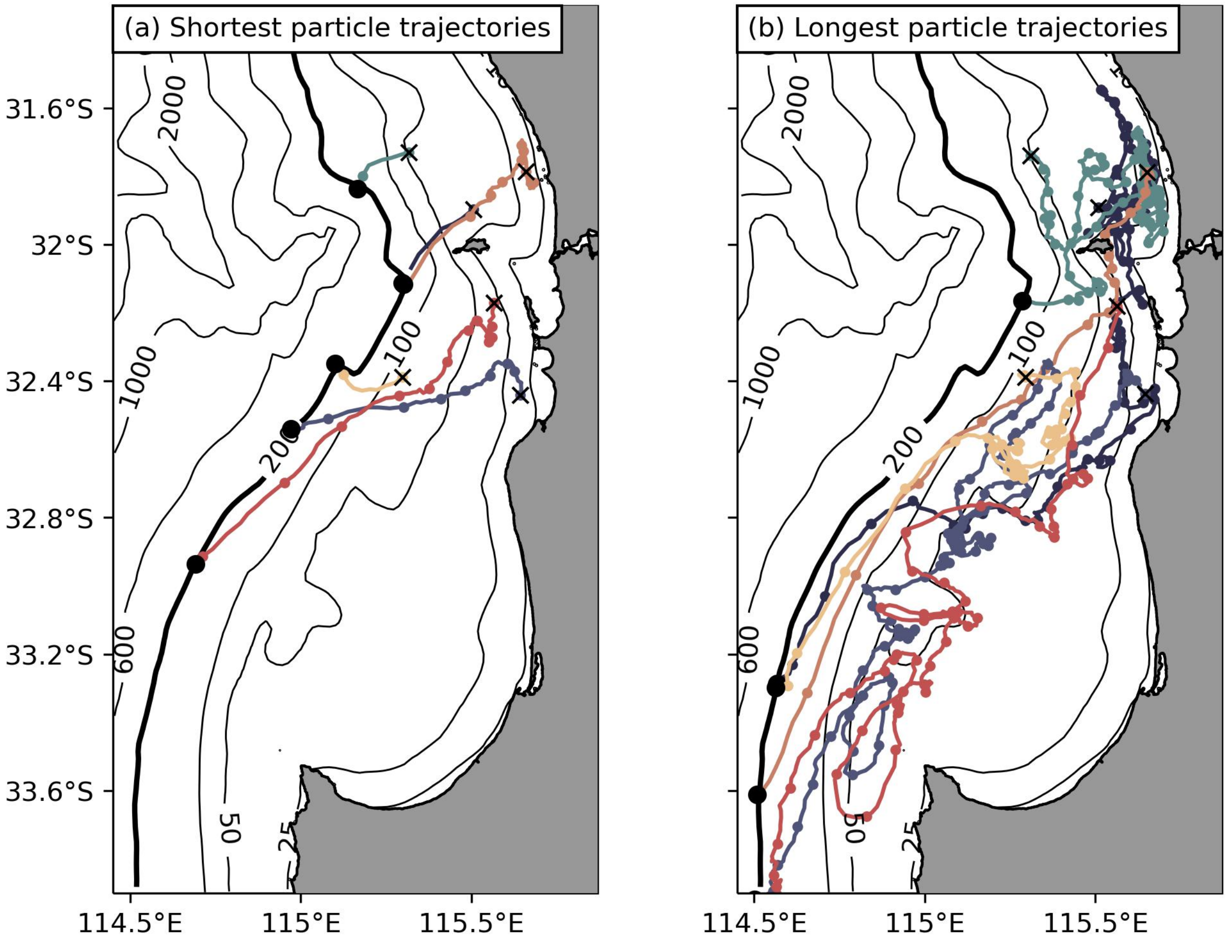

Figure S12 Example trajectories of simulated kelp detritus being transported past the continental shelf edge (black dot indicates where each particle crosses the shelf edge) released from six different locations (black crosses). The trajectories shown are those of particles that took the **(a)** shortest; and **(b)** longest time to cross the continental shelf edge (of all particles that were transported past the shelf edge from that pixel). The dots on each trajectory are spaced 1 day apart to give an indication of how fast particles were moving in each location.
